# Modeling analysis of depolarization-assisted afterdischarge in hippocampal mossy fibers

**DOI:** 10.3389/fncir.2024.1505204

**Published:** 2025-01-08

**Authors:** Haruyuki Kamiya

**Affiliations:** Department of Neurobiology, Hokkaido University Graduate School of Medicine, Sapporo, Japan

**Keywords:** axon, ectopic burst, hippocampus, mossy fiber, simulation

## Abstract

A strong repetitive stimulus can occasionally enhance axonal excitability, leading to the generation of afterdischarge. This afterdischarge outlasts the stimulus period and originates either from the physiological spike initiation site, typically the axon initial segment, or from ectopic sites for spike generation. One of the possible mechanisms underlying the stimulus-induced ectopic afterdischarge is the local depolarization due to accumulated potassium ions surrounding the axonal membranes of the distal portion. In this study, the mechanisms were explored by computational approaches using a simple model of hippocampal mossy fibers implemented with the structure of *en passant* axons and experimentally obtained properties of ionic conductances. When slight depolarization of distal axons was given in conjunction with the high-frequency stimulus, robust afterdischarges were triggered after cessation of the repetitive stimulus and lasted for a prolonged period after the stimulus. Each spike during the afterdischarge recorded from distal axons precedes that recorded from the soma, suggesting that the afterdischarge was ectopically generated from distal axons and propagated antidromically toward the soma. Notably, when potassium channels in the model are replaced with non-inactivating ones, repetitive stimuli fail to induce afterdischarge. These results suggested that the inactivating property of axonal potassium channels plays a crucial role in generating the afterdischarge. Accumulated inactivation of potassium channels during strong repetitive stimulation may alter mossy fiber excitability, leading to ectopic afterdischarges from sites distinct from the physiological spike initiation region.

## Introduction

Neurons are thought to be an analog integrator of excitation and inhibition from multiple inputs converging onto them. Once the membrane potential exceeds the threshold membrane potentials, action potentials are triggered from the spike initiation sites located at the proximal portions of axons (Schmidt-Hieber et al., 2008) or the axon initial segments (Meeks and Mennerick, 2007a; Ohura and Kamiya, 2016; Alpizar et al., 2019). In addition to the canonical mode of spike signaling along axons, it has been suggested that a non-canonical mode called retroaxonal action potentials is triggered from axons distal from the soma in both physiological and pathological conditions (Gutnick and Prince, 1972; Stasheff and Wilson, 1990; Pinault, 1995; Kapoor et al., 1997; Amir et al., 2005; Bucher and Goaillard, 2011; Bähner et al., 2011; Sheffield et al., 2011; Dugladze et al., 2012; Thome et al., 2018; Liu et al., 2022; Kramer et al., 2022). Massive neuronal activity often has an impact to drastically raise neuronal excitability, which causes the non-canonical mode of signaling in the brain, namely ectopic firings from different sites of physiological spike initiation, in an activity-dependent manner (Stasheff and Wilson, 1990; Stasheff et al., 1993; Sheffield et al., 2011). High-frequency activity has been shown to lead to retroaxonal barrage firings of some central neurons such as cortical inhibitory neurons (Sheffield et al., 2011; Suzuki et al., 2014). Induction mechanisms involve astrocytes (Meeks et al., 2005; Deemyad et al., 2018), hyperpolarization-activated cyclic-nucleotide-gated (HCN) channels (Elgueta et al., 2015; Rózsa et al., 2023; see also Roth and Hu, 2020), and K^+^ accumulation by the repetitive stimulus (Smith, 1983; Meeks and Mennerick, 2004, 2007b; Foley et al., 2010; Beswick-Jones et al., 2023; Rózsa et al., 2023). It was found that the hippocampal mossy fiber also displays stimulus-induced prolonged hyperexcitability accompanying the enhancement of spontaneous discharges (Kamiya, 2017), although the mechanisms remain obscure since the hippocampal mossy fiber axons do not significantly express HCN channel subtypes HCN1-4 (Notomi and Shigemoto, 2004). To test for the involvement of membrane depolarization caused by K^+^ accumulation surrounding the axons by the repetitive stimulus in the stimulus-induced afterdischarge, the computational approaches using a NEURON simulation environment were adopted in this study (Hines and Carnevale, 1997). Using a simple and realistic model of hippocampal mossy fiber with the structure of the *en passant* axons implemented with conductance characterized by the direct electrophysiological recordings from the axonal boutons (Engel and Jonas, 2005; Ohura and Kamiya, 2018), the conditions indispensable to generating stimulus-induced bursts were explored. It was found that a repetitive stimulus with depolarization of distal axons to −70 mV elicited persistent afterdischarge lasting after cessation of the stimulus. The afterdischarge was supposed to be generated ectopically from the distal axons, different from the physiological spike initiation site at the proximal axons or the axon initial segment, suggested by the reversal of the order of the somatic and the axonal spikes after the induction of the afterdischarge. Intriguingly, the stimulus-induced afterdischarge was abolished when the K^+^ channel model was replaced with a non-inactivating one. Taken together, our simulation data suggested that inactivating properties of K^+^ channels are involved in the depolarization-assisted generation of afterdischarge originating from the ectopic site from the physiological generating site of action potentials at the hippocampal mossy fiber synapse.

## Materials and methods

### Simulation

Computer simulations of axonal membrane potentials (V_m_) were performed using NEURON 8.2 for Windows (Hines and Carnevale, 1997). In brief, the structure of the mossy fiber (Acsády et al., 1998; Henze et al., 2000) was approximated by a soma (diameter, 10 μm), 11 axonal cylinders (diameter, 0.2 μm; length, 100 μm), and 10 *en passant* boutons (diameter, 4 μm). The number of segments was 1 μm^−1^ in all simulations. For the majority of simulations in this study, the time step was set at 0.1 ms to describe the fast action potential kinetics and the underlying currents. In some simulations for calculating the time derivative of membrane potential (dV_m_/dt), the time step was set at 0.01 ms to accurately trace dV_m_/dt. The passive electrical properties of the axon were assumed to be uniform, with a specific membrane capacitance C_m_ of 1 μF cm^−2^, a specific membrane resistance R_m_ of 10,000 Ω cm^2^, and an intracellular resistivity R_i_ of 110 Ω cm (Engel and Jonas, 2005; Hallermann et al., 2003). The resting membrane potential was set at −80 mV unless otherwise stated.

Voltage-gated Na^+^ channels, K^+^ channels, and leakage channels were inserted into the soma, axon, and boutons, as described by the previous study (Kamiya, 2019). The Na^+^ conductance density was set at 50 mS cm^−2^ for the axon and boutons and 10 mS cm^−2^ for the soma. The K^+^ conductance density was set at 36 mS cm^−2^ throughout all parts of the neurons. Action potentials were evoked by the injection of depolarizing current into the soma (2 ms, 0.2 nA). The equilibrium potentials for Na^+^ and K^+^ ions were assumed to be +50 and −85 mV, respectively. In some simulations, the non-inactivating K^+^ channel model, which lacks inactivation properties, was used instead of the inactivating K^+^ channel model. The hhmfb.mod, a Hodgkin–Huxley type model for the set of sodium, potassium, and leakage channels adapted to channels in mossy fiber terminals, was downloaded from ModelDB (accession no. 128079) for a non-inactivating I_K_ model for sodium and potassium conductances. For an inactivating I_K_ model, the potassium conductance in hhmfb.mod was omitted and replaced with KIn.mod, a Hodgkin–Huxley model of inactivating K channels representing the kinetics of K_V_1.4 (Wissmann et al., 2003), which was downloaded from the same ModelDB (accession no. 128079).

**Figure 1:**
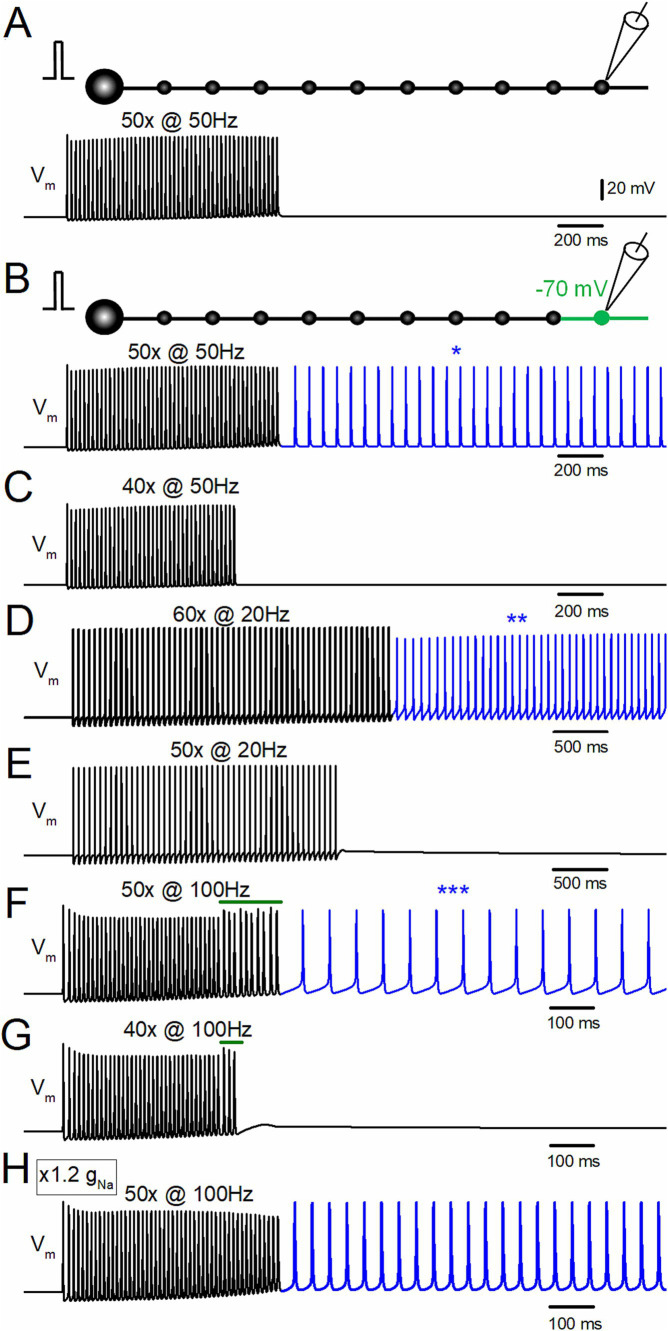
Depolarization-assisted induction of afterdischarge by repetitive stimulation of the hippocampal mossy fiber model. **(A)** Effect of repetitive stimulation of 50 times at 50 Hz at a resting potential of −80 mV. Membrane potential (V_m_) at the 10th bouton is shown. **(B)** When the distal portion of the axon (the 10th bouton and the axons on both sides, shown in *green* in the schematic drawing) was depolarized to −70 mV, the same repetitive stimulation evoked afterdischarge persistent after the termination of the repetitive stimulus, as shown in the *blue trace* and an asterisk (*). **(C)** Depolarization-assisted afterdischarge was not induced with the stimulus of 40 times at 50 Hz with the same local depolarization. **(D)** The stimulus at 20 Hz applied 60 times, with slight depolarization of the distal axon to −70 mV also induced persistent afterdischarge (*blue trace*, **). **(E)** The stimuli at 20 Hz applied 50 times did not induce burst firings. **(F)** Stimulus-induced afterdischrge was also induced by 100 Hz stimuli 50 times (*blue trace*, ***). **(G)** The stimulus at 100 Hz applied 40 times is insufficient for generating afterdischarge. **(H)** Effect of 100 Hz stimuli applied 50 times to the model whose g_Na_ value was increased from the original value of 50 mS cm^−2^ to 60 mS cm^−2^. All the results suggest that local small depolarization is sufficient for assisting in boosting regenerative afterdischarge.

Action potentials at the mossy fiber boutons, as well as the granule cell somata evoked by the repetitive action potentials of 50 times at 50 Hz, 60 times at 20 Hz, and 50 times at 100 Hz, were calculated for testing the use-dependent initiation of ectopic afterdischarge of the mossy fiber axon. To check for the threshold of stimulation number required for burst generation, stimuli of 40 times at 50 Hz, 50 times at 20 Hz, and 40 times at 100 Hz were also tested, as shown in Figure 1. For local depolarization, the resting membrane potential was changed by altering the el value (equilibrium potential of leak conductance). All model and simulation files will be available from the ModelDB database as accession no. 2018003.^1^

## Results

### Depolarization-assisted induction of afterdischarge by repetitive stimulation of the mossy fiber model

To test for the possible induction of stimulus-induced hyperexcitability of the axon, a repetitive stimulus of 50 times at 50 Hz was applied to the model of mossy fiber axons. In the normal condition at the resting membrane potentials at −80 mV (Engel and Jonas, 2005), repetitive stimulation of 50 times at 50 Hz did not induce persistent discharges after the termination of the repetitive stimulus (Figure 1A). In contrast, with slight depolarization of the distal part of the axon (the 10th bouton to −70 mV), the same repetitive stimulus elicited persistent firings after cessation of the repetitive stimulus (Figure 1B). The frequency of the afterdischarge induced by 50 Hz 50 times stimuli was 15.8 Hz. This stimulus-induced burst shows a regenerative and paroxysmal nature in its generation (Coggan et al., 2010) since a shorter repetitive stimulus of 40 times at 50 Hz did not elicit the burst discharge (Figure 1C). Even when the stimulus frequencies are changed to 20 Hz (Figures 1D,E) and 100 Hz (Figures 1F,G), similar results were obtained, i.e., repetitive stimuli induced persistent firings after the termination of the stimulus train above the threshold stimulus number. For example, 60 times at 20 Hz stimuli with depolarization to −70 mV induced the stimulus-induced afterdischarge of 14.7 Hz (Figure 1D), and 50 times at 100 Hz with the same depolarization elicited afterdischarge of 16.1 Hz (Figure 1F). The frequency of afterdischarge did not vary much among the stimulus frequencies of 20, 50, and 100 Hz. Shorter repetitive stimuli of 50 times at 20 Hz (Figure 1E) or 40 times at 100 Hz (Figure 1G) did not induce persistent firings after the termination of the repetitive stimulus. Notably, the action potentials during the 100 Hz stimulus were suppressed slightly at the initial phase and showed irregularity of the amplitudes at the later phase (*green bars* in Figures 1F,G). The initial decrease may be caused by incomplete recovery from the inactivation of Na^+^ channels since lower frequency stimulus (20–50 Hz) did not cause the depression of action potentials. The irregular responses at the late phase are caused by propagation failures that appear only at the late phase of the repetitive stimuli. One possible explanation is that accumulated fractional inactivation of sodium channels or insufficient recovery from inactivation of voltage-dependent sodium channels at short intervals (10 ms) reduced the available sodium channels and caused propagation failures. In support of this notion, it was found that the propagation failure was abolished if the g_Na_ value was increased from the original value of 50 mS cm^−2^ to 60 mS cm^−2^, as shown in Figure 1H. These findings suggest that a small local depolarization is sufficient to boost regenerative afterdischarge originating from an ectopic spike initiation site in the axon.

### Antidromic propagation of the stimulus-induced afterdischarge of the mossy fiber model

**Figure 2:**
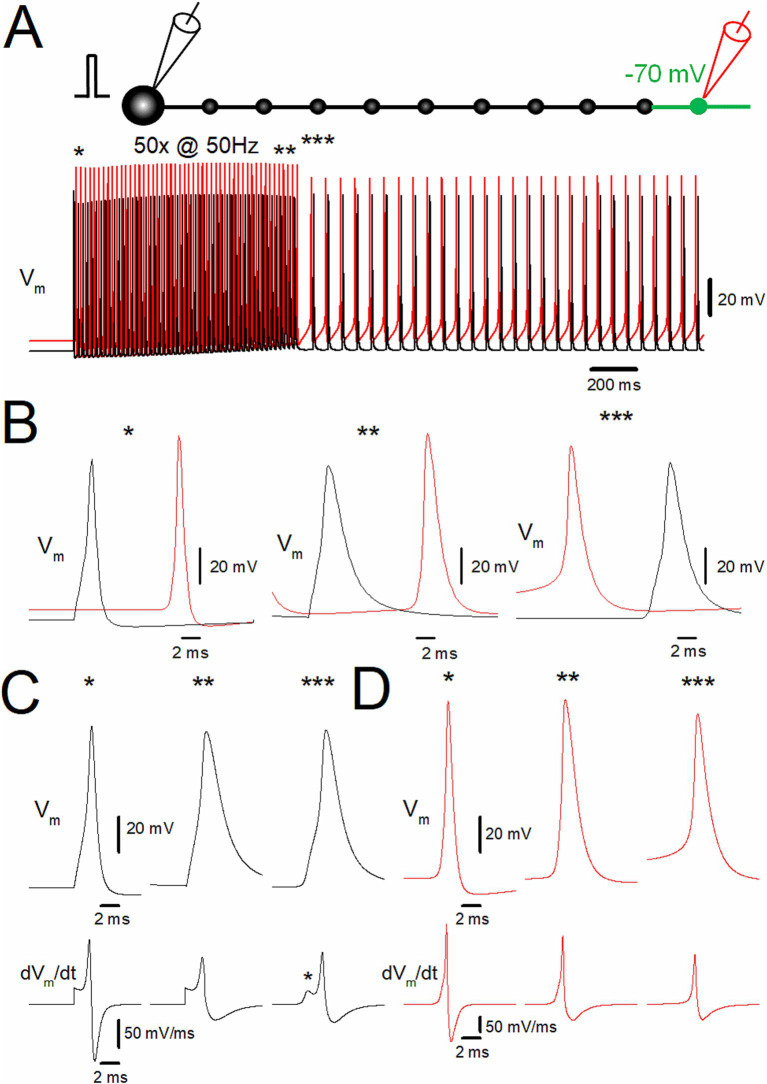
Ectopic spiking of the distal axon during depolarization-assisted stimulus-induced afterdischarge. **(A)** Stimulus-induced afterdischarge induced by repetitive stimulation of 50 times at 50 Hz with slight depolarization of distal axon to −70 mV, as shown in Figure 1B. Membrane potentials (V_m_) at the soma and the 10th bouton are shown in *black* and *red traces*, respectively. **(B)** The time-expanded traces of somatic (*black trace*) and axonal action potentials (*red trace*) at the first (*; left), the last (**; middle), and after (***; right) the repetitive stimulus. **(C)** Comparison of the action potential kinetics (V_m,_ upper traces) and the time derivative of V_m_ (dV_m_/dt, lower traces) at the soma (*black traces*). The asterisk (*) shows IS-SD inflections characteristic of antidromic spikes. **(D)** The similar data set of V_m_ at the 10th bouton (*red traces*) as in panel **(C)**. The data for **(C)** and **(D)** are collected by the simulations with a faster sampling rate at 100 kHz to trace dV_m_/dt with sufficient temporal resolution. These data suggest ectopic origin and subsequent antidromic propagation of stimulus-induced afterdischarge.

Since the depolarization was applied to the distal portion of the axon in the previous simulation, it was hypothesized that the afterdischarge was triggered from the depolarized part of the distal axon and propagated antidromically to the soma. To test this hypothesis, the membrane potentials at the soma (*black traces*) and the 10th bouton (*red traces*) were calculated (Figure 2A). Both at the first (*) and the last action potentials (**) during the repetitive stimulus of 50 times at 50 Hz, somatic action potentials (*black traces*) were followed by those at the 10th bouton (*red trace*), indicating that action potentials were generated near the soma and propagated orthodontically toward the distal axons (Figure 2B, left and middle). In contrast, during the stimulus-induced persistent afterdischarge (***) following the repetitive stimulation, however, the order was reversed as *red* to *black* sequence (Figure 2B, right), as expected for the ectopic site for the burst firings from the depolarized portions of the axon and subsequent antidromic propagation toward the soma. Antidromic propagation of the stimulus-induced burst was also supported by the different action potential kinetics during and after the repetitive stimulus (Figures 2C,D). For traces in Figures 2C,D, simulations were performed at a faster sampling rate of 100 kHz to accurately monitor the kinetics of membrane potential (V_m_) and its time derivative (dV_m_/dt). The action potential kinetics was slower during the stimulus-induced afterdischarge. The half-duration of the somatic action potentials at the first (*) and the last (**) of the repetitive stimulus was 1.11 ms and 2.69 ms, respectively, while it was 2.40 ms after the repetitive stimulus for 50 times at 50 Hz. A slower action potential kinetics was also observed for the 10th bouton (Figure 2D). The half-duration of the action potentials at the 10th bouton during the same repetitive stimulus was 0.86 ms and 1.69 ms, respectively, while it was 1.53 ms after the repetitive stimulus. Taken together, all the evidence so far supported the generation of stimulus-induced afterdischarge from the ectopic site at the distal axons whose membrane potentials were slightly depolarized. The ectopic afterdischarge was propagated antidromically back to the soma. It should be noted that it is evident that the inflection of the waveform of the first derivative of the somatic membrane potential (dV_m_/dt) exists in the rising phase. This inflection (asterisk in Figure 2C) may represent IS-SD inflection (Eccles et al., 1966; Stasheff et al., 1993), which may be caused by the impedance mismatch of the small initial segment (IS) and a large volume of somatodendritic compartment (SD) and thus supposed to indicate the antidromic invasion of axonal spikes to the soma after the generation of the ectopic afterdischarge from the distal axon. All the findings are in support of the antidromic propagation of the stimulus-induced afterdischarge of the mossy fiber model.

### Inactivating the property of K^+^ channels assists the stimulus-induced afterdischarge of the mossy fiber model

**Figure 3:**
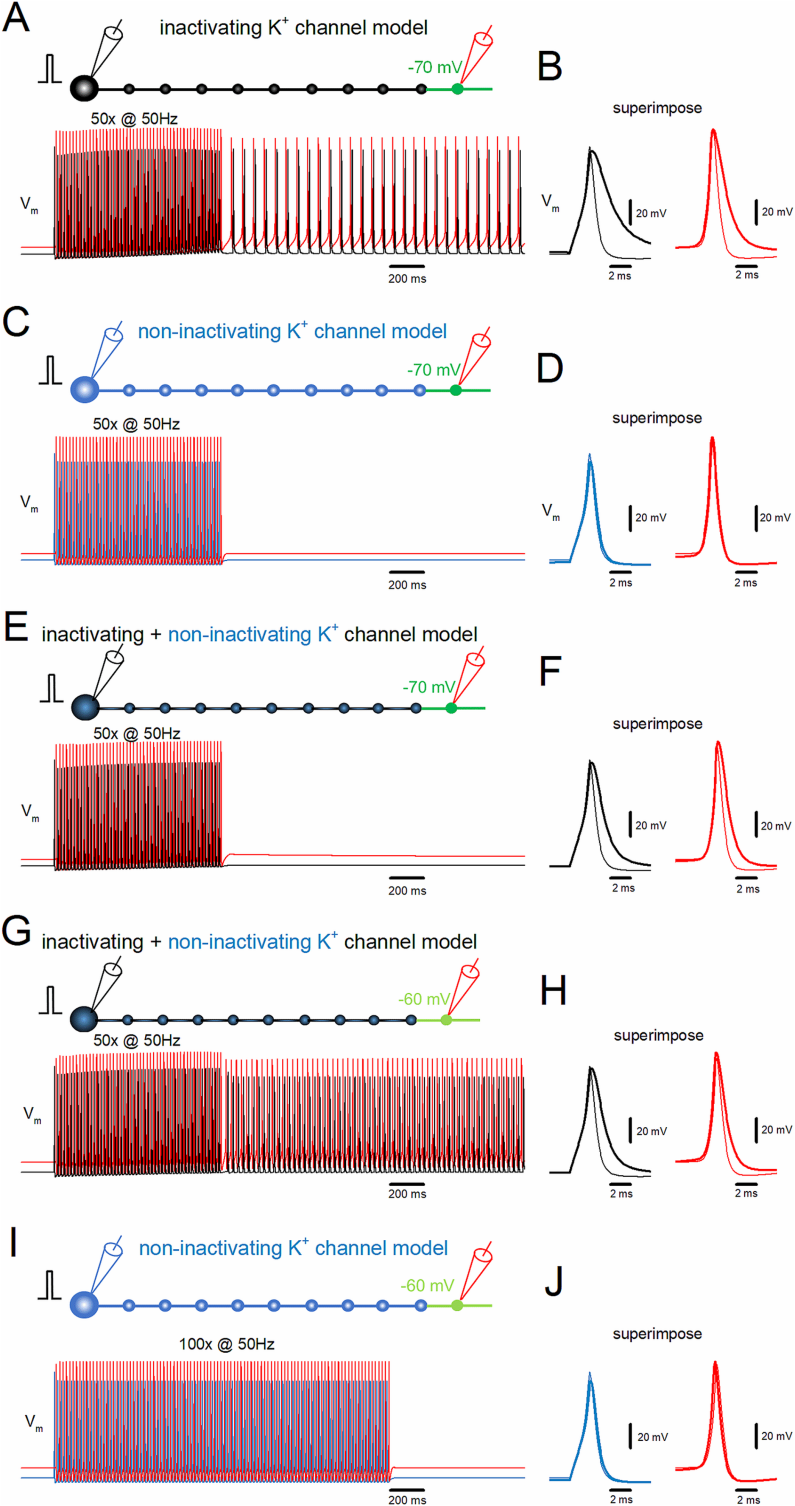
Inactivation of K^+^ channels assists induction of the stimulus-induced afterdischarge. **(A)** Simulation in the same conditions as in Figure 2A. **(B)** The time-expanded traces of action potentials (V_m_) at the soma (*black trace*) and distal axon (*red trace*). The first (thin traces) and the last action potentials (thick traces) during the repetitive stimulus are superimposed. **(C)** Similar data as in panel **(A)** except that the model of K^+^ channels was replaced with those removed inactivation (non-inactivating K^+^ channel model, *blue trace*). **(D)** Similar data as in panel **(B)** with the non-inactivating type K^+^ channel model. **(E,F)** The model of K^+^ channels was replaced with a mixture of 90% inactivating type and 10% non-inactivating type and displayed as **(A)** and **(B)**. **(G,H)** Similar data as in panels **(E)** and **(F)** when the distal portion of the axon was depolarized to −60 mV. **(I,J)** Simulation with longer repetitive stimulus (50 Hz 100 times) with strong depolarization of the distal portion to −60 mV with the non-inactivating K^+^ channel model. All the results suggest that the accumulated inactivation of K^+^ channels during the repetitive stimulus assists the generation of the afterdischarge in conjunction with local axonal depolarization of the distal axon.

Action potentials propagating along the hippocampal mossy fibers display use-dependent modification of the duration by slowing the falling phase (Geiger and Jonas, 2000). This unique property of axonal spike signaling imparts additional computing ability for fine-tuning the network function. As for the mechanisms, it was demonstrated that accumulated inactivation of K^+^ channels during the repetitive action potentials was critically involved (Geiger and Jonas, 2000; see also Zheng and Kamiya, 2023). It was inferred by analogy that the inactivation of K^+^ channels may underlie the generation of the stimulus-induced afterdischarge of the hippocampal mossy fibers. To test for the contribution of the inactivating property of axonal K^+^ channels, the effects of removal of inactivation from the axonal K^+^ channel model were explored (Zheng and Kamiya, 2023). A repetitive stimulation applied 50 times at 50 Hz induced the stimulus-induced afterdischarge, which outlasted the repetitive stimulus (Figure 3A). During the repetitive stimulation, action potentials both at the soma (*black traces*) and the 10th bouton (*red traces*) prolonged the kinetics by slowing the falling phase (Figure 3B). The half-duration of the first somatic action potential during the repetitive stimulus was 1.10 ms (*black thin trace*), while the half-duration of the last (50th) action potential was 2.68 ms (*black thick trace*). At the 10th bouton, the half-duration of the first action potentials during the repetitive stimulus was 0.85 ms (*red thin trace*), while the half-duration of the 50th action potential was 1.68 ms (*red thick trace*). These findings confirmed the results of our previous exploration (Zheng and Kamiya, 2023).

In contrast, if the model of axonal K^+^ channels was replaced with those without inactivation (Zheng and Kamiya, 2023), the stimulus-induced burst was not induced (Figure 3C). In this condition, action potentials do not display use-dependent broadening during the repetitive stimulation of 50 times at 50 Hz (Figure 3D). The half-duration of the first somatic action potential during the repetitive stimulus was 1.10 ms (*blue thin trace*), while the half-duration of the 50th action potential was 1.27 ms (*blue thick trace*). At the 10th bouton, the half-duration of the first action potentials was 0.88 ms (*red thin trace*), while the half-duration for the 50th action potential was 0.90 ms during the repetitive stimulus (*red thick trace*). All the results are consistent with the notion that the accumulated inactivation of K^+^ channels during the repetitive stimulus assists the generation of the stimulus-induced afterdischarge of the mossy fiber model in conjunction with local small depolarization of the distal axon.

Although mossy fiber boutons predominantly express inactivating K^+^ channels (Geiger and Jonas, 2000), multiple types of K^+^ channels have been identified that shape action potentials, including some that do not inactivate (Alle et al., 2011). To investigate whether stimulus-induced afterdischarge could occur with a mixture of inactivating and non-inactivating channels, a model combining 90% inactivating and 10% non-inactivating channels was tested. The same depolarization of the distal portions to the −70 mV did not induce the afterdischarge (Figure 3E), although action potentials displayed a partial broadening of duration (Figure 3F). The half-duration of the first somatic action potential during the repetitive stimulus was 1.14 ms (*black thin trace*), while the half-duration of the last (50th) action potential was 1.91 ms (*black thick trace*). At the 10th bouton, the half-duration of the first action potential during the repetitive stimulus was 0.88 ms (*red thin trace*), while the half-duration of the 50th action potential was 1.14 ms (*red thick trace*). With this combination of inactivating and non-inactivating K^+^ channels, a stimulus-induced afterdischarge was elicited when the depolarization of the distal portions was increased to −60 mV, as shown in Figure 3G. The afterdischarge frequency was 31.7 Hz. The half-duration of the first somatic action potential during the repetitive stimulus was 1.14 ms (*black thin trace*), while the half-duration of the last (50th) action potential was 1.91 ms (*black thick trace*). At the 10th bouton, the half-duration of the first action potential during the repetitive stimulus was 0.88 ms (*red thin trace*), while the half-duration of the 50th action potential was 1.29 ms (*red thick trace*) (Figure 3H). These results confirmed the causal relationships between the inactivation properties of K^+^ channels and the generation of stimulus-induced afterdischarge.

One may argue that non-inactivating K^+^ channels may simply raise the threshold, but not be indispensable, for the generation of afterdischarge. To address this point, an additional simulation was performed to apply longer repetitive stimuli of 100 times at 50 Hz with the depolarization of distal portions to −60 mV. Even though this condition was expected to enhance the possibility of generating afterdischarge, the repetitive stimuli did not induce afterdischarge (Figure 3I) nor broadening of action potentials (Figure 3J). Taken together, the inactivation of axonal K^+^ channels is critically important for the stimulus-induced afterdischarge at the hippocampal mossy fibers.

### Stimulus-induced ectopic afterdischarge is triggered along the course of mossy fibers

**Figure 4:**
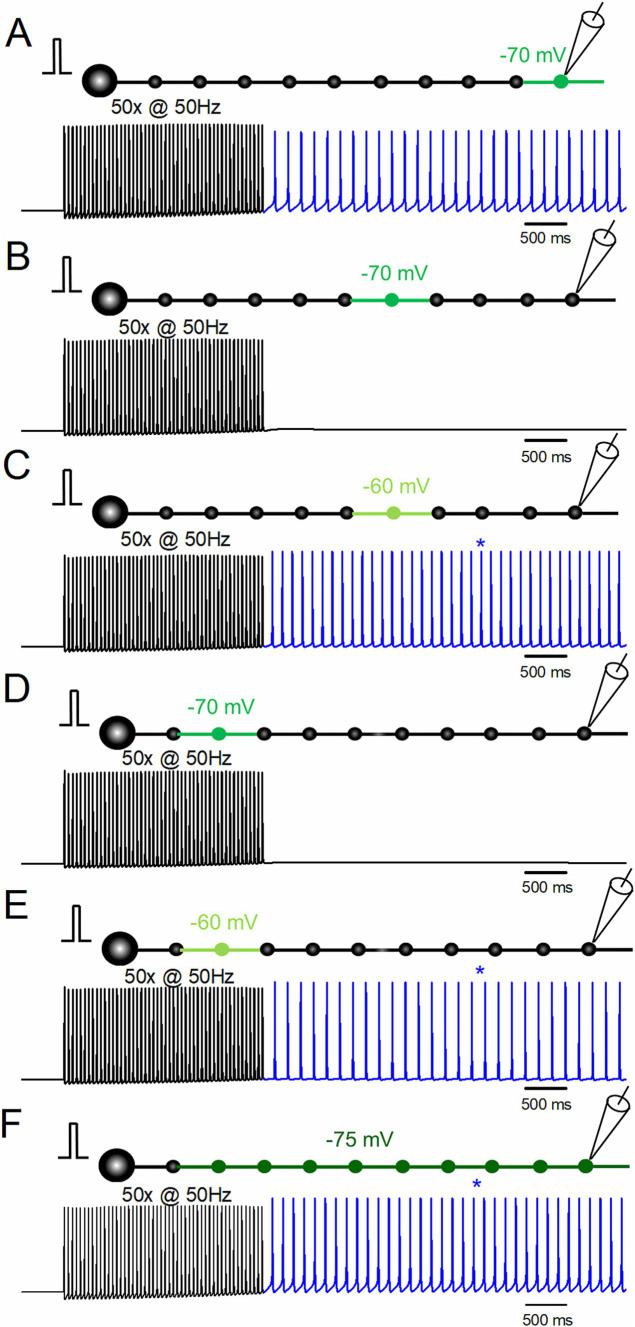
Effects of the location of depolarization on stimulus-induced afterdischarge. **(A)** Simulation in the same conditions as in Figure 1B. **(B,C)** Responses to repetitive stimulation of 50 times at 50 Hz with slight depolarization of the middle portion of the axon (the 6th bouton and the axons on both sides) to −70 mV **(B)** and −60 mV **(C)**. **(D,E)** Simulation with depolarization of the proximal portion of the axon (the 2nd bouton and the axons on both sides) to −70 mV **(D)** and −60 mV **(E)**. **(F)** Simulation with uniform depolarization of the axon (the 2nd–10th boutons and the axons on both sides) to −75 mV. The results suggest that depolarization-assisted generation of the stimulus-induced afterdischarges initiated from the whole axon.

To date, the role of local depolarization of distal portions of the mossy fiber axons has been explored, as shown in Figure 4A. To further confirm the robustness of the notion that local depolarization assisted the generation of ectopic afterdischarge, the effects of the depolarization of the distal, middle, and proximal portions of mossy fiber axons and the uniform depolarization of the course of mossy fiber axons were explored. When the middle portion (the sixth boutons and both side axons) was depolarized to −70 mV, the stimulus-induced afterdischarge was not elicited (Figure 4B), while the same repetitive stimulation of 50 times at 50 Hz caused afterdischarge when depolarization was increased to −60 mV (Figure 4C). The frequency of the afterdischarge was 20.0 Hz. Similar results were obtained when the proximal portion (the second boutons and both side axons) was depolarized to −70 mV (Figure 4D) or to −60 mV (Figure 4E). The frequency of the afterdischarge was 15.5 Hz. It should be noted that the uniform depolarization of the axons (the 2nd–10th boutons and the axons on both sides) also elicited stimulus-induced afterdischarge with weaker depolarization to −75 mV (Figure 4F). The frequency of the afterdischarge was 19.2 Hz. These results suggest that depolarization-assisted generation of the stimulus-induced afterdischarge can be initiated from the whole axon.

In this series of simulations, local depolarization was given to the portion of the axon by changing the equilibrium potential of the leak conductance. It should be noted that the steady-state level of depolarization did not reach the value since the depolarized compartment was connected to the non-depolarized compartment. To explore the threshold membrane potentials for the generation of afterdischarge, the steady-state membrane potentials at the depolarized compartment were calculated as −74.5 mV, −75.7 mV, −72.9 mV, −76.0 mV, −73.1 mV, and −75.3 mV for the simulation conditions of Figures 4A–F, respectively. It might be intriguing to speculate that the threshold for generating afterdischarge lies near −75 mV, although this is a too simplified notion not considering complex spatiotemporal dynamics of the axonal excitability. More importantly, it implicates the physiological significance that local small depolarization may cause a drastic impact to shift to the non-canonical mode of axonal signaling.

### Bi-directional propagation of the stimulus-induced burst of the mossy fiber model

**Figure 5:**
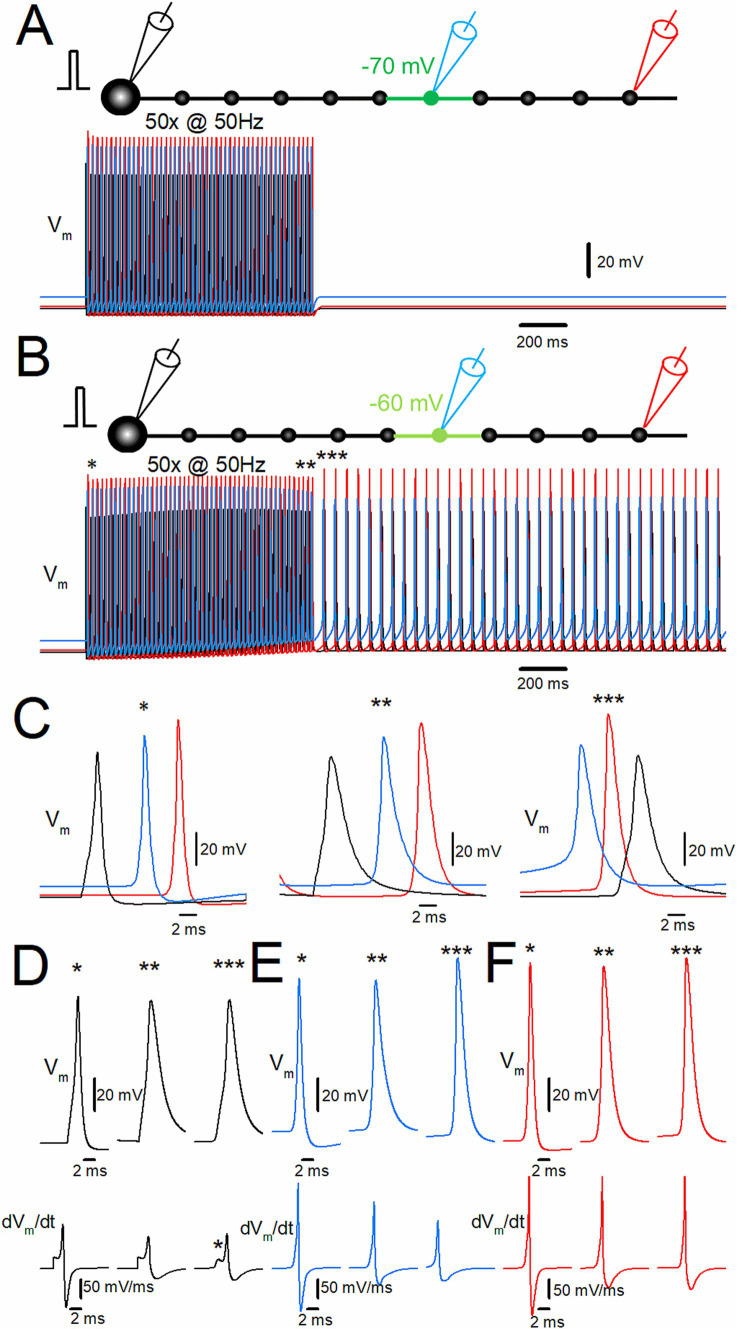
Bi-directional propagation of the afterdischarge from the site of local depolarization. **(A)** Effect of repetitive stimulation of 50 times at 50 Hz with depolarization of the 6th bouton and the axons on both sides to −70 mV. Membrane potentials (V_m_) at the soma, the 6th bouton, and the 10th bouton are shown in *black*, *blu*e, and *red traces*, respectively. **(B)** When the 6th bouton and the axons on both sides, shown in *green* in the schematic drawing, were depolarized to −60 mV, stimulus-induced afterdischarge was induced after cessation of the repeated stimulus. **(C)** The time-expanded traces of action potentials at the soma (*black trace*), the 6th bouton (*blue trace*), and the 10th bouton (*red trace*) at the first (*; left), the last (**; middle), and after (***; right) the repetitive stimulus. **(D)** Comparison of the action potential kinetics (V_m_, upper traces) and the time derivative of V_m_ (dVm/dt, lower traces) at the soma (*black traces*). **(E,F)** The similar data set of V_m_ at the 6th bouton (*blue traces*) and 10th bouton (*red traces*) as in panel **(D)**. The data for **(D–F)** are collected by the simulations with a faster sampling rate at 100 kHz to trace dV_m_/dt with sufficient temporal resolution. The results suggest bi-directional propagation of the afterdischarge from the site of initiation.

To date, the effects of local small depolarization in conjunction with the repetitive stimulus of the granule cells were examined, and it was found that the stimulus-induced burst discharges with ectopic origin were triggered after the repetitive stimulus and propagated antidromically to the soma. To explore the possible orthodromic propagation of the ectopic burst, repetitive stimulus for 50 times at 50 Hz in conjunction with depolarization to −70 mV of the sixth bouton and the axons on both sides did not induce the afterdischarge by the repetitive stimulus, as shown in Figure 5A. In contrast, the same repetitive stimulus with depolarization of the same compartments to −60 mV triggered the afterdischarge by the repetitive stimulus, as shown in Figure 5B. To determine the order of action potentials at the soma, the 6th bouton, and the 10th bouton, time-expanded traces were analyzed, as shown in Figure 5C. During the stimulus train (left and middle traces, * and **), the action potential at the soma (*black trace*) was followed by the action potential at the 6th (*blue trace*) and the 10th boutons (*red trace*), consistent with the orthodromic propagation from the soma to the distal axon. On the other hand, during the stimulus-induced afterdischarge, the order of these action potentials changed. The action potential at the 6th bouton where local depolarization was applied (*blue trace*) was followed by the action potentials at the 10th bouton (*red trace*) and the soma (*black trace*), as shown in the right traces of Figure 5C (***). It should be noted that the action potential kinetics at the soma were different during and after the repetitive stimulus (Figure 5D). The half-duration of the somatic action potentials at the first (*) and the last (**) of the repetitive stimulus was 1.11 ms (*) and 2.68 ms (**), respectively. After 50 repetitions at 50 Hz (***), the half-duration was 2.44 ms after the repetitive stimulus. A slower action potential kinetics was also observed for the 6th (Figure 5E) and the 10th boutons (Figure 5F). The half-duration of the action potentials at the 6th bouton at the first (*) and the last (**) of the repetitive stimulus was 0.85 ms and 1.85 ms, respectively, while it was 1.84 ms after the repetitive stimulus (**). The half-duration of the action potentials at the 10th bouton at the first (*) and the last (**) of the repetitive stimulus was 0.88 ms and 1.73 ms, respectively, while it was 1.62 ms after the repetitive stimulus (**). The different action potential kinetics during and after the repetitive stimulus were consistent with the notion that the stimulus-induced afterdischarge by the repetitive stimuli was triggered ectopically at different sites from the physiological spike initiation and propagated bi-directionally following the ectopic afterdischarge generation at the depolarized portions.

## Discussion

In this study, a numerical simulation approach was adopted to examine the mechanisms underlying the stimulus-induced afterdischarge at the hippocampal mossy fiber, which does not substantially express HCN channels, key molecules for this form of plasticity of excitability of axons. A repetitive stimulus, in conjunction with a small depolarization of distal portions of the mossy fiber, triggered a prolonged afterdischarge in the simple biophysical model of mossy fiber. The stimulus-induced burst firings were supposed to be generated at ectopic sites of spike initiation different from the physiological spike initiation sites, due to the reversed order of action potentials at the soma and the distal axon and the different waveform of action potentials during and after the repetitive stimulus. Depolarization-assisted stimulus-induced bursts would be an additional mechanism for the retroaxonal barrage firings observed experimentally.

### Stimulus-induced burst firings induced in the mossy fiber model

Recent studies have shown that strong repetitive stimulation occasionally triggers afterdischarge persistent even after the cessation of the repetitive stimulus in some neurons. Some cortical inhibitory neurons have been shown to exhibit robust plasticity in the intrinsic excitability of their axons (Sheffield et al., 2011). This form of plasticity of the axonal excitability called retroaxonal barrage firings is facilitated by the HCN channels on the axons (Elgueta et al., 2015; Rózsa et al., 2023; see also Roth and Hu, 2020) and K^+^ accumulation by the repetitive stimulus (Smith, 1983; Meeks and Mennerick, 2004, 2007b; Foley et al., 2010; Beswick-Jones et al., 2023; Rózsa et al., 2023) or surrounding glia (Meeks et al., 2005; Deemyad et al., 2018). Repeated stimulation of the mossy fiber has been demonstrated to induce the stimulus-induced prolonged repeated firings (Kamiya, 2017), although the mechanisms remain uncertain since the hippocampal mossy fiber axons do not substantially express HCN channel subtypes HCN1-4, which has been shown to mediate the induction of burst discharges (Notomi and Shigemoto, 2004). A recent study has indicated that a local increase in potassium concentration also promoted the induction of stimulus-induced afterdischarge in the cortical inhibitory interneurons (Rózsa et al., 2023). In analogy with this observation, I wondered if local subthreshold depolarization, assuming a local increase in potassium concentration, can assist the induction of the bursts. As expected from this notion, it was confirmed that local depolarization of the axonal portion to −70 mV from the resting membrane potential of −80 mV can generate stimulus-induced afterdischarge, as shown in Figure 1. The paroxysmal nature was demonstrated by the finding that the stimulus numbers above the threshold are needed for the induction of the stimulus-induced burst, as also shown in Figure 1.

### Ectopic origin of stimulus-induced burst firings from distal axon

Regarding the nature of the stimulus-induced afterdischarge, it was noted that the prolonged repeated discharges were generated from the distal axon where small depolarization was applied. This contrasts with the action potential initiation from the proximal site of the mossy fiber (Schmidt-Hieber et al., 2008) in the physiological condition. Emerging evidence suggests that the action potentials are generated not only from the proximal axon or the axon initial segments but also from the distal axon ectopically (Stasheff et al., 1993; Avoli et al., 1998; Sheffield et al., 2011; Dugladze et al., 2012). As in these experimental findings, the stimulus-induced afterdischarge was triggered with the assistance of depolarization of the distal portion of the axon. The ectopic origin of the afterdischarge in the hippocampal mossy fibers is supported by several lines of the findings of this study. First, the stimulus-induced bursts are triggered only when small depolarization is applied to distal portions of the axon which is far from the physiological spike initiation site at the proximal axon (Schmidt-Hieber et al., 2008). Second, the action potentials at the distal axon were followed by those at the soma during the bursts, indicating antidromic propagation of the burst firings, as shown in Figure 1. Third, the somatic action potential during the stimulus-induced bursts displayed the inflection on the rising phase, resembling the IS-SD inflection characteristic for invasion of antidromic propagation to the soma (Eccles et al., 1966; Stasheff et al., 1993), as shown in Figure 2C. These findings are all consistent with the ectopic origin of the stimulus-induced afterdischarge and the subsequent antidromic propagation toward the soma.

Since the simulation also indicates that the ectopically triggered afterdischarge propagates bi-directionally from the site of burst generation, as shown in Figure 5, the stimulus-induced ectopic bursts are supposed to cause the massive transmitter release and a barrage of postsynaptic currents on the postsynaptic CA3 pyramidal neurons and lead to raising the excitatory tones in the CA3 neuronal network (Suzuki et al., 2014). On the other hand, however, antidromic invasion of bursting action potentials may collide with and therefore suppress output signals from the soma. Synaptic plasticity caused by the stimulus-induced bursts (Bukalo et al., 2013) also needs to be considered for the net influence of the burst firings. Future investigations should determine the spatiotemporal consequences of stimulus-induced bursts on activity within the CA3 neuronal network.

### Use-dependent broadening of action potentials assists induction of the stimulus-induced bursts

A characteristic feature of hippocampal mossy fibers is the activity-dependent broadening of action potentials, caused by the progressive slowing of the falling phase due to the cumulative inactivation of voltage-dependent potassium channels (Geiger and Jonas, 2000; see also Zheng and Kamiya, 2023). By removing the inactivation of the axonal K^+^ channels in models of computer simulations, the depolarization-assisted stimulus-induced burst firings were not induced in the same repetitive stimulation, as shown in Figure 4. It would be interesting to address this notion experimentally, although no pharmacological tools to selectively and completely suppress the inactivation of voltage-dependent K^+^ channels are available currently. Future studies are needed to test this idea in experiments.

In this study, a series of numerical simulations using a simple model of hippocampal mossy fiber was performed to test for the possible induction of the stimulus-induced burst firings persisting even after the termination of the repetitive stimulus. The simulation demonstrated that the repetitive stimulation with the aid of local small depolarization of the axon generates regenerative burst firings in the model. Ectopic generation of afterdischarge was implicated by the signs of antidromic propagation including reversed order of somatic and axonal action potentials and IS-SD inflection at the rising phase of the somatic action potentials. Use-dependent broadening of action potentials by accumulated inactivation of K^+^ channels has been suggested to mediate the generation of the stimulus-induced afterdischarge. Taking all pieces of evidence into account, these mechanisms are suggested to promote this form of plasticity of axonal excitability of hippocampal mossy fiber axons.

## Data Availability

The raw data supporting the conclusions of this article will be made available by the authors without undue reservation.

## Footnotes

^1^ https://modeldb.science/2018003
